# Hydrogen Flux Inhibition of Pd-Ru Membranes under Exposure to NH_3_

**DOI:** 10.3390/membranes14030059

**Published:** 2024-02-25

**Authors:** Lingsu Chen, Shuai Li, Zhaohui Yin, Zhanbing Yang, Zihui Chen, Li Han, Qinghe Yu, Miao Du

**Affiliations:** 1 China GRINM Group Co., Ltd., National Engineering Research Center of Nonferrous Metals Materials and Products for New Energy, Beijing 100088, China; 2 GRIMAT Engineering Institute Co., Ltd., Beijing 101407, China; 3 General Research Institute for Nonferrous Metals, Beijing 100088, China; 4 School of Metallurgical and Ecological Engineering, University of Science and Technology Beijing, Beijing 100083, China

**Keywords:** Pd-Ru membrane, hydrogen flux, NH_3_ exposure, poisoning mechanism

## Abstract

The hydrogen flux inhibition of Pd-Ru membranes under exposure to 1–10% NH_3_ at 673–773 K was investigated. The Pd-Ru membranes were characterized by XRD, SEM, XPS, and hydrogen permeation tests. The results show that when exposed to 1–10% NH_3_ at 723 K for 6 h, the hydrogen flux of Pd-Ru membranes sharply decreases by 15–33%, and the decline in hydrogen flux becomes more significant with increasing temperatures. After the removal of 1–10% NH_3_, 100% recovery of hydrogen flux is observed. XPS results show that nitrogenous species appear on the membrane surface after NH_3_ exposure, and the hydrogen flux inhibition may be related to the competitive adsorption of nitrogenous species. By comparing the hydrogen flux of Pd-Ru membranes exposed to 10% NH_3_ with 10% N_2_, it is indicated that the rapid decrease in hydrogen flux is due to the concentration polarization and competitive adsorption of nitrogenous species. The competitive adsorption effect is attenuated, while the concentration polarization effect becomes more pronounced with increasing temperature.

## 1. Introduction

In recent decades, hydrogen as an ideal energy carrier has received wide attention due to its CO_X_-free nature, environmental friendliness, and high energy density [1]. However, the difficulty in hydrogen storage and transportation and the poor safety hinder the further application of hydrogen. At present, large-scale storage and transportation of hydrogen are mainly achieved with compressed hydrogen [2] and liquid hydrogen [3], but both methods have the limitations of large energy consumption. Alternatively, hydrogen can be stored by chemical storage using hydrogen carriers such as methanol, formic acid, NH_3_, etc. NH_3_ is liquid at room temperature and medium pressure (<1 MPa) [4], and it is relatively easy to transport and store. The hydrogen storage density of NH_3_ (17.8 wt.% [5]) surpasses that of methanol (12.6 wt.% [6]) and formic acid (4.34 wt.% [7]), making it a promising liquid hydrogen carrier. Hydrogen can be produced by the decomposition of NH_3_, followed by hydrogen separation and purification to meet the requirements of downstream applications. This offers a practical solution for the efficient and secure storage, delivery, and versatile usage of hydrogen.

The gases produced by thermal decomposition of NH_3_ are hydrogen, nitrogen, and trace ammonia. Hydrogen needs to be further separated and purified from these decomposition gases for downstream applications. Traditional hydrogen purification methods like pressure swing adsorption (PSA) have drawbacks such as high energy consumption, low hydrogen purity, and large foot-print area [8]. Hydrogen purification using Pd membranes shows great potential in small- to medium-scale hydrogen production [9,10]. By integrating the NH_3_ decomposition process with membrane purification in a single chamber [11,12], the separation and purification of hydrogen are realized while the decomposing reaction is conducted. This facilitates the transition of NH_3_ decomposition reaction towards the product side, overcoming the thermodynamic constraints of the NH_3_ decomposition reaction [13], significantly reducing its operating temperature (<773 K) and improving the NH_3_ conversion efficiency. Cechetto et al. [14] found that the NH_3_ decomposition membrane reactor achieved 15% higher NH_3_ conversion than conventional reactors. The hydrogen yield exceeded 86%, and the purity of hydrogen reached 99.998%. This demonstrates the potential of membrane reactors in NH_3_ decomposition for hydrogen production.

In NH_3_ decomposition membrane reactors, Pd-based membranes, such as pure Pd [15,16], Pd-Ag [13,17], Pd-Au [18], etc., exhibit outstanding performance in terms of hydrogen separation. However, pure Pd membranes have limitations in terms of working temperature. The pure Pd membranes may cause hydrogen embrittlement at low temperatures (<573 K). Although the above-mentioned Pd-Ag and Pd-Au alloy membranes show property improvements in the aspects of hydrogen embrittlement and hydrogen permeation, defects or pinholes develop with time at high temperatures, and they have poor resistance to impurity poisoning. Pd-Ru membranes were widely used in membrane reactions for their high hydrogen permeability, resistance against impurity, and high thermal stability [19,20,21].

In the process of NH_3_ decomposition, the presence of N_2_ and residual NH_3_ may adversely affect the hydrogen permeation performance of Pd membranes. Sakamoto et al. [22] investigated the effects of N_2_ and NH_3_ on the hydrogen permeation performance of Pd membranes. The hydrogen flux of pure Pd membranes was reduced by 13% under exposure to 10% NH_3_ at 523 K, while it was only reduced by 9% after exposure to 10% N_2_. They observed the N 1s spectra by XPS on the membrane after NH_3_ exposure, and the decrease in hydrogen flux was mainly due to the competitive adsorption on the membrane surface occupying the hydrogen dissociation adsorption site. However, the XPS analysis showed that chemical species, such as NH_2_, NH_3_, –NO, NO_2_, etc., could not be identified. Zhang et al. [23] showed a slight decrease (6.3%) in the hydrogen flux of pure Pd membranes with increasing NH_3_ content in the feed gas from 920 ppm to 63,000 ppm, and no significant hydrogen permeability change was observed over several days. However, owing to the change in hydrogen content at different temperatures, it remains uncertain whether hydrogen partial pressure or NH_3_ contributes to the decreased hydrogen flux. Similarly, Lundin et al. [24] did not observe any inhibition of hydrogen fluxes by N_2_ or NH_3_ at 673 K and 1 MPa. To evaluate the effect of trace NH_3_, Peters et al. [25] investigated the flux inhibition of Pd_77_Ag_23_ membranes exposed to 10–500 ppm NH_3_. They found no inhibition effect on the hydrogen flux of Pd_77_Ag_23_ membranes when exposed to 200 ppm NH_3_ at 723 K. However, the hydrogen flux of Pd_77_Ag_23_ membranes was reduced by 26% in the presence of 200 ppm NH_3_ at 573 K. They concluded that the inhibition of hydrogen flux might be related to the incorporation of changes in the Pd alloy membranes due to surface segregation.

It is clear that the reported effects of NH_3_ on the hydrogen flux of Pd-based membranes are contradictory, and the poisoning mechanism of NH_3_ remains unclear. The role of NH_3_ seems more complex, and specific studies on individual factors are needed to further elucidate their poisoning mechanisms. In particular, a few issues about the poisoning of NH_3_ on Pd-Ru membranes are discussed. To further improve the overall performance of Pd-Ru membranes and better optimize the operating conditions in membrane reactors, it is essential to clarify the inhibition effect of NH_3_ on the hydrogen flux of Pd-Ru membranes under the conditions of NH_3_ decomposition.

In this paper, we investigate the hydrogen permeation performance of Pd-Ru membranes exposed to 1–10% NH_3_ at 673–773 K and different pressures. We present the effects of NH_3_ concentration and exposure temperatures on the hydrogen flux of Pd-Ru membranes, and the NH_3_ poisoning mechanism on the Pd-Ru membranes is analyzed.

## 2. Materials and Methods

### 2.1. Membrane Preparation

Porous alumina ceramic tubes with a diameter of 12 mm and an average pore size of 0.05 μm were used as the supports of the Pd-Ru membrane. The Pd-Ru membrane was deposited by a sequential electroless plating technique [26]. The surface of the porous alumina ceramic tubes was uniformly sprayed with palladium acetate dissolved in chloroform. Subsequently, the tubes were heat-treated for 3 h at 523 K in an air atmosphere to convert the palladium acetate into palladium oxide. These heat-treated substrate tubes were then reduced in hydrazine baths for 1 h to form palladium nano-particles. The Pd layer was deposited on the activated surface by electroless plating at 308 K, followed by the deposition of the Ru layer on top of the Pd layer at 333 K. The bath compositions of the electroless plating are presented in Table 1. The as-deposited Pd-Ru membranes were heat-treated by a hydrogen permeation process at 723 K for 24 h.

### 2.2. NH_3_ Poisoning Tests and Characterization

Graphite seals were used to seal the Pd-Ru membrane tubes into tubular stainless steel cavities. The feed gases entered through the shell side and the hydrogen permeated through the membrane to the tube side. The feed pressure was controlled with a pressure regulator installed on the retentate side.

Prior to NH_3_ exposure, pure hydrogen permeation tests were performed on the Pd-Ru membranes in the range of 623–773 K and 0.1–0.4 MPa. NH_3_ poisoning tests were performed by passing a 1–10% NH_3_/H_2_ mixture through these membranes for 6 h at 723 K. After NH_3_ exposure, pure hydrogen was introduced to evaluate the recovery performance of hydrogen flux. Pd-Ru membranes exposed to 10% NH_3_ were further examined for changes in hydrogen flux under different temperatures and pressures. For comparative analysis, the NH_3_ poisoning tests were all performed on the same membrane. And different membrane samples prepared with the same preparation process were used in the post-process characterization.

The surface microstructural characterization of the membranes was performed by using a Hitachi S-4800 scanning electron microscope (SEM, Hitachi Limited, Tokyo, Japan). The phase and composition of the membranes before and after NH_3_ exposure were determined using a Rigaku D/max 2500 X-ray diffractometer (XRD, Rigaku Corporation, Tokyo, Japan) and ESCA-LAB Xi+ X-ray photoelectron spectroscopy (XPS, Thermo Fisher Scientific, Waltham, MA, USA). For the XPS analysis, the C 1s reference signal was used as a calibrated reference.

## 3. Results and Discussion

### 3.1. Hydrogen Permeation Experiments

**Figure 1:**
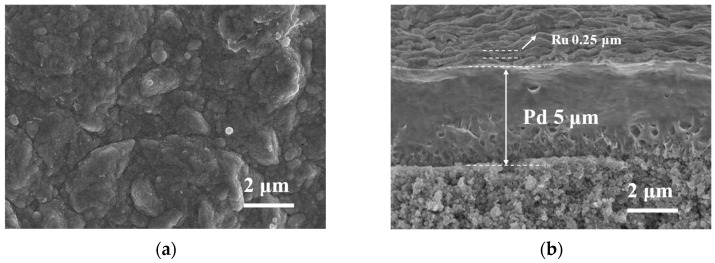
SEM images of the surface (**a**) and cross-section (**b**) of the as-deposited Pd-Ru membrane.

**Figure 2:**
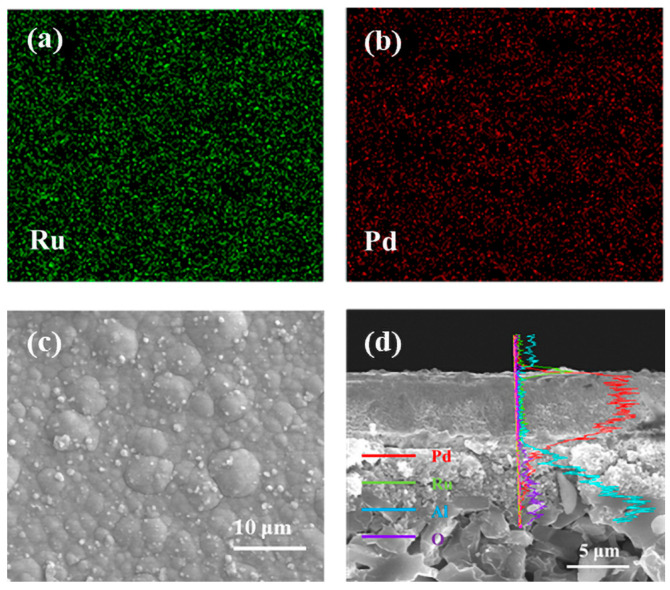
EDS analysis of the Pd-Ru membrane: (**a**) EDS surface Pd mapping; (**b**) EDS surface Ru mapping; (**c**) SEM surface image; (**d**) EDS line scans.

SEM micrographs of the as-deposited Pd-Ru membrane are shown in Figure 1. The membrane is compact without pinholes or cracks, which confirms that the electroless plating conditions used in this study are suitable for preparing a uniform membrane surface. As shown in Figure 1b, the Pd-Ru membrane has two distinct layers, Pd and Ru layers. The thickness of the Pd layer is about 5 μm, and the thickness of the Ru layer is about 0.25 μm. EDS mappings of the Pd-Ru membrane are shown in Figure 2, and it is clear that the Ru layer is above the Pd layer. According to the thickness of the Pd-Ru membrane and the metal density, the Ru content is calculated as about 5 wt.%.

**Figure 3:**
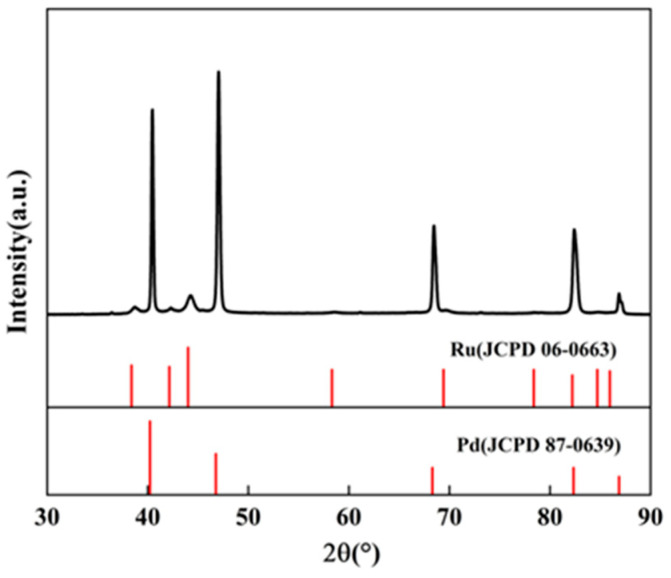
XRD pattern of the as-deposited Pd-Ru membrane.

The XRD pattern of the as-deposited Pd-Ru membrane is shown in Figure 3. The diffractogram exhibits peaks of the Pd phase and Ru phase, indicating that the Pd-Ru membrane has been successfully prepared.

Hydrogen permeation through Pd membranes follows the solution–diffusion mechanism [27]. Driven by the partial pressure difference between the two sides of the membrane, hydrogen diffuses from the high-pressure region to the low-pressure region. Hydrogen molecules adsorbed on the surface of the Pd membrane undergo dissociation into hydrogen atoms, which then dissolve in the Pd lattice. When diffused to the membrane surface of the low-pressure side, hydrogen molecules are reconnected and desorbed from the Pd surface. The hydrogen permeation flux is obtained by combining Fick and Sieverts’ law:

(1) $$J_{H_{2}}=\frac{P_{e}}{d}[{\left(P_{1}\right)}^{n}-{\left(P_{2}\right)}^{n}],$$

where $J_{H_{2}}$ is the hydrogen permeate flux of the membrane, mol∙m^−2^∙s^−1^; *P_e_* is the hydrogen permeability of the membrane, mol∙m^−1^∙s^−1^∙Pa^−*n*^; *d* is the total thickness of the membrane, m; *P*_1_ is the partial pressure of hydrogen on the feed side, Pa; *P*_2_ is the partial pressure of hydrogen on the permeated side, Pa; and *n* is the pressure exponent.

**Figure 4:**
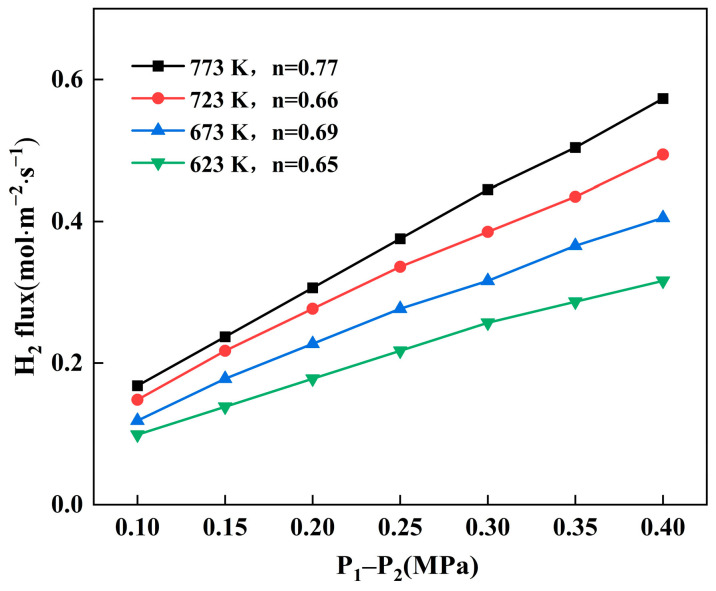
Hydrogen permeation flux of the Pd-Ru membrane as a function of pressure difference at different temperatures. *n* is the pressure exponent.

Figure 4 shows the variation in hydrogen flux through Pd-Ru membranes at different temperatures and pressures. The hydrogen flux gradually increases with the rising temperature and pressure difference. At temperatures of 623 K, 673 K, 723 K, and 773 K, the hydrogen permeability values for the Pd-Ru membrane are measured as follows: 4.2 × 10^−9^ mol∙m^−1^∙s^−1^∙Pa^−0.5^, 5.3 × 10^−9^ mol∙m^−1^∙s^−1^∙Pa^−0.5^, 6.4 × 10^−9^ mol∙m^−1^∙s^−1^∙Pa^−0.5^, and 7.3 × 10^−9^ mol∙m^−1^∙s^−1^∙Pa^−0.5^, respectively. The determined value agreed quite well with values reported in the literature [28]. The pressure exponent *n* values are measured as follows: 0.65, 0.69, 0.66, and 0.77. The values of the pressure exponent *n* are all in the range of 0.5–1, indicating that both the surface reaction and the bulk diffusion of the hydrogen atoms control the hydrogen permeation through the Pd-Ru membranes. At a temperature of 723 K, the H_2_/N_2_ selectivity value for the Pd-Ru membrane at 0.1 MPa is 1960.

### 3.2. Effect of NH_3_ on Hydrogen Permeation through Pd-Ru Membrane

#### 3.2.1. Effect of NH_3_ Exposure Concentration

**Figure 5:**
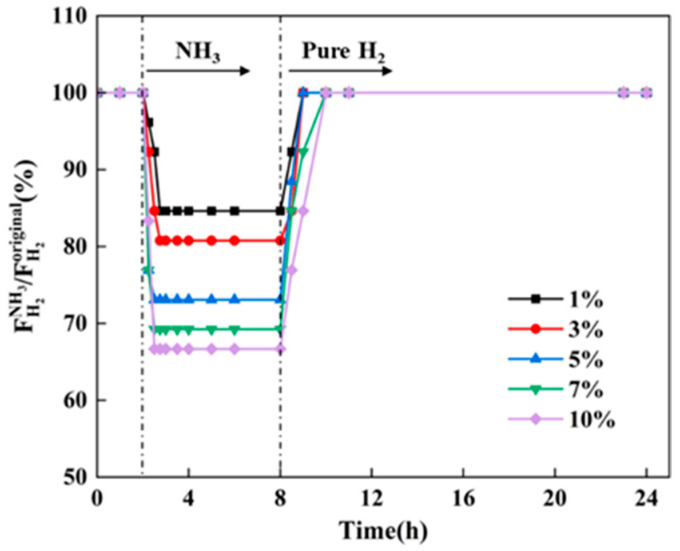
Hydrogen flux inhibition of Pd-Ru membranes exposed to different concentrations of NH_3_ at 723 K and 0.1 MPa for 6 h and the subsequent recovery in pure hydrogen.

Figure 5 shows the variation in relative hydrogen flux of Pd-Ru membranes exposed to 1–10% NH_3_ at 0.1 MPa for 6 h at 723 K and the subsequent recovery in pure hydrogen. The above test conditions are based on the conditions of NH_3_ decomposition, which were convenient to compare with literature data. ”The relative hydrogen flux ($F_{H_{2}}^{{\mathrm{NH}}_{3}}/F_{H_{2}}^{\mathrm{original}}$) is the ratio of the hydrogen flux of the Pd-Ru membrane exposed to NH_3_ ($F_{H_{2}}^{{\mathrm{NH}}_{3}}$) to the original hydrogen flux ($F_{H_{2}}^{\mathrm{original}}$) before NH_3_ exposure.

When exposed to 1–10% NH_3_, the relative hydrogen fluxes through Pd-Ru membranes decreased sharply by 15–33% before rapidly stabilizing at new steady-state values. The inhibition effect of NH_3_ became more pronounced with the increase in NH_3_ concentration. After NH_3_ removal, the hydrogen fluxes all recovered to 100%, indicating that the inhibition caused by NH_3_ on the hydrogen fluxes is completely reversible. The physical adsorption of NH_3_ on the surface of Pd-Ru membranes may be a reason for the decrease in hydrogen fluxes. This is mainly due to the fact that among the available adsorption sites, the top side of the horizontal orientation for hydrogen molecules exhibits the most energetically favorable condition (−0.343 eV), while the adsorption energy of NH_3_ molecules is greater than −0.84 eV, slightly stronger than that of hydrogen [29,30]. The recovery time of hydrogen flux after pure hydrogen treatment increases with increasing NH_3_ concentration. These occupied active sites are gradually released over time following pure hydrogen treatment, resulting in the recovery of hydrogen flux. Thus, it can be inferred that the decrease in hydrogen flux may be related to the physical adsorption of NH_3_ on the surface of the Pd-Ru membrane.

#### 3.2.2. Effect of NH_3_ Exposure Temperature

**Figure 6:**
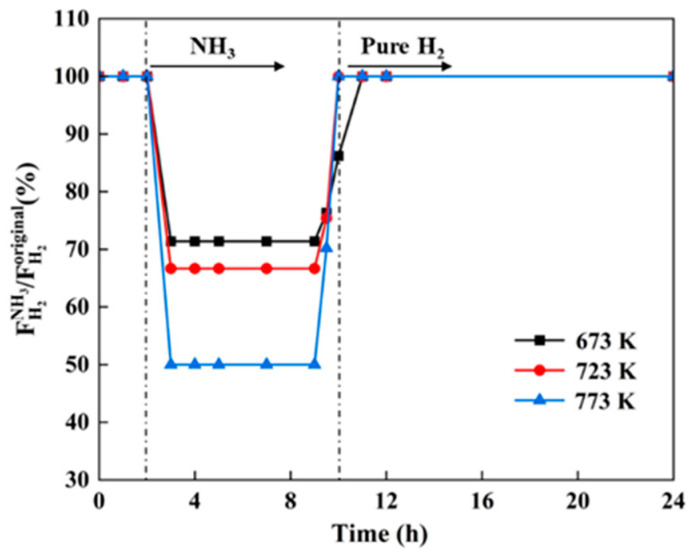
Hydrogen flux inhibition of the Pd-Ru membrane exposed to 10% NH_3_ at 0.1 MPa for different temperatures and the subsequent recovery in pure hydrogen.

As mentioned above, the inhibition effect of 10% NH_3_ on the hydrogen flux of the Pd-Ru membrane is more significant. Therefore, 10% NH_3_ was chosen to investigate the effect of NH_3_ at different exposure temperatures. Figure 6 shows the relative hydrogen flux of Pd-Ru membranes exposed to 10% NH_3_ at various temperatures at 0.1 MPa and the subsequent recovery using pure hydrogen. The hydrogen flux of Pd-Ru membranes rapidly decreased by 27–50% after exposure to 10% NH_3_ at 673–773 K, and the inhibition extent of NH_3_ increased with increasing temperature. All hydrogen fluxes recovered to 100% upon introducing pure hydrogen.

In addition to competitive adsorption effects, the concentration polarization caused by the accumulation of non-permeable gases on upstream surfaces also blocks the permeation of hydrogen. For the NH_3_/H_2_ mixtures, both physical adsorption and concentration polarization effects may occur. N_2_ is usually regarded as an inert gas, and hydrogen flux inhibition is observed after the introduction of N_2_. This is typically attributed to the concentration polarization rather than competing adsorption [24]. To comprehend the effects of concentration polarization and competitive adsorption of NH_3_, the variation in the hydrogen flux through Pd-Ru membranes exposed to 10% NH_3_ and 10% N_2_ at different temperatures was investigated.

**Figure 7:**
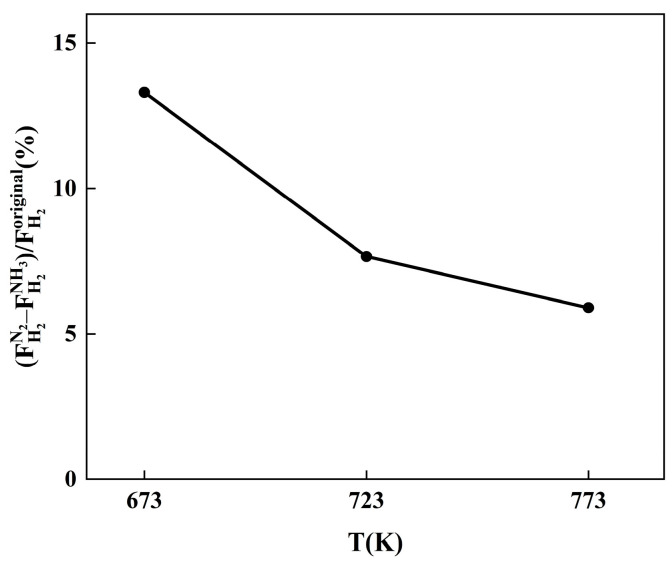
Relative hydrogen flux difference for Pd-Ru membranes exposed to 10% NH_3_ and 10% N_2_ at 0.1 MPa and different temperatures.

Figure 7 shows the temperature dependence of relative hydrogen flux for the Pd-Ru membrane when exposed to 10% NH_3_ and 10% N_2_ at 673–773 K. The difference in relative hydrogen flux ($(F_{H_{2}}^{N_{2}}–F_{H_{2}}^{{\mathrm{NH}}_{3}})/F_{H_{2}}^{\mathrm{original}}$) represents the competitive adsorption effect of NH_3_. The permeation of H_2_/N_2_ can be used to study concentration polarization. The decrease in hydrogen flux increases with rising temperature when exposed to 10% N_2_, which proves a gradual increase in concentration polarization effect. There are two main effects of the temperature on concentration polarization [31]. On the one hand, higher temperature enhances the diffusion coefficient and improves the mass transfer behavior, thereby weakening the concentration polarization effect. On the other hand, with higher temperatures, the hydrogen flux through the membrane is enhanced and the concentration polarization effect becomes more severe. As shown in Figure 6, with increasing temperature from 673 to 773 K, the difference in the relative hydrogen flux between Pd-Ru membranes exposed to 10% NH_3_ and 10% N_2_ decreased from 13.3% to 5.9%, indicating a gradual reduction in the competitive adsorption effect. The results suggest that both concentration polarization and competitive adsorption of NH_3_ occur simultaneously when the Pd-Ru membrane is exposed to NH_3_. Competitive adsorption has a bigger effect at lower temperatures, whereas concentrated polarization becomes more prominent at higher temperatures. The combined effect of these two effects ultimately leads to an increase in the inhibition effect of NH_3_ with rising temperature, as shown in Figure 5.

**Figure 8:**
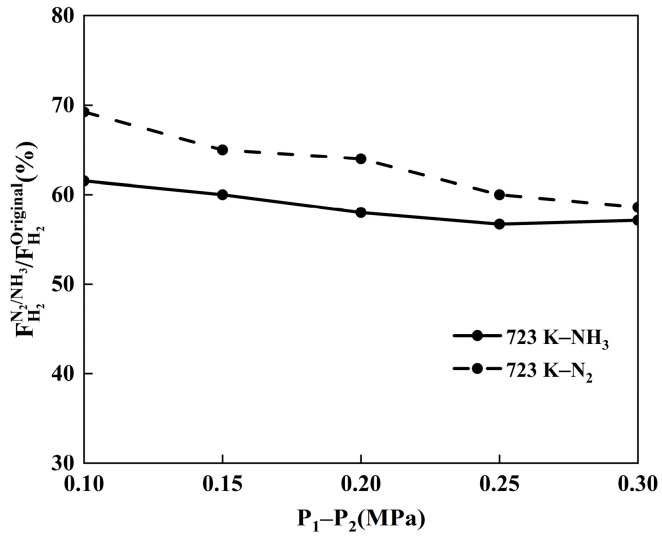
Variation in relative hydrogen permeation flux with pressure for Pd-Ru membranes exposed to 10% NH_3_ and 10% N_2_ at 723 K.

Figure 8 shows the variation in hydrogen flux with pressure when the Pd-Ru membrane is exposed to 10% NH_3_ and 10% N_2_ at 723 K. Compared with N_2_, the inhibition of hydrogen flux is more pronounced for the same NH_3_ concentration, further indicating that the poisoning effect of NH_3_ on Pd-Ru membranes is influenced by other factors in addition to the concentration polarization effect. With the same N_2_ concentration, there is a slight increase in hydrogen flux inhibition with increasing pressure due to an elevation in pressure leading to a higher hydrogen composition gradient and a lower diffusion coefficient. As a result, the effect of concentration polarization increases with rising pressure. In contrast, there are no significant changes in the hydrogen flux under NH_3_ exposure at different pressures, indicating that pressure plays a minor role in the NH_3_ poisoning for Pd-Ru membranes.

### 3.3. Post-Process Characterization

**Figure 9:**
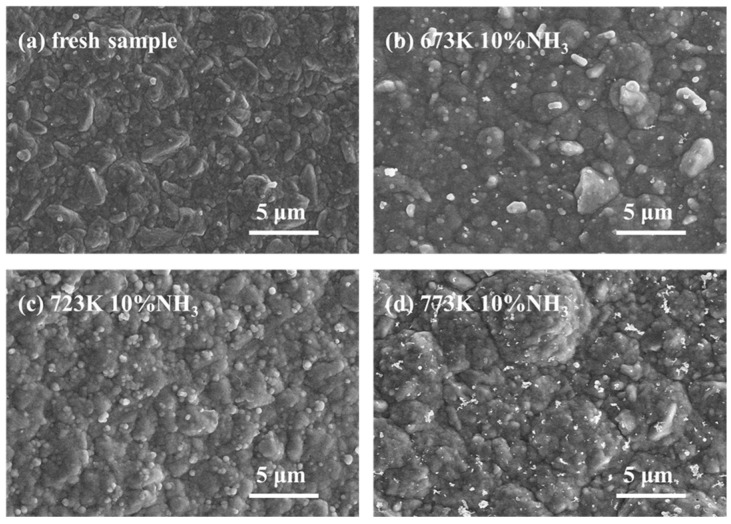
SEM images of Pd-Ru membranes exposed to 10% NH_3_ at different temperatures: (**a**) fresh sample; (**b**) 673 K; (**c**) 723 K; (**d**) 773 K.

Figure 9 shows the SEM images of Pd-Ru membranes after exposure to 10% NH_3_ for 6 h at various temperatures. A surface smoothening and grain size growth are observed after exposure to 10% NH_3_. No cracks or pinholes are observed on the Pd-Ru membranes. The N_2_ flux of the Pd-Ru membrane before and after NH_3_ exposure did not show any significant changes and the Pd-Ru membranes remained dense and defect-free, indicating that NH_3_ exposure has no significant effect on the surface morphology of the Pd-Ru membranes.

**Figure 10:**
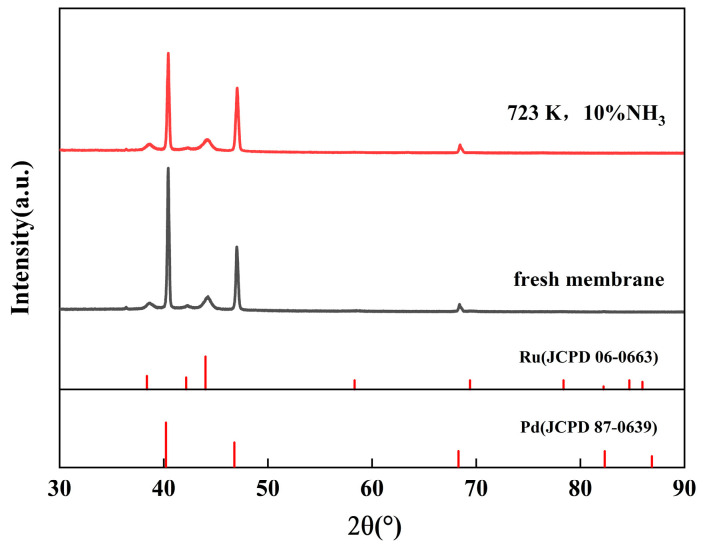
Grazing incidence XRD pattern of Pd-Ru membranes after exposure to 10% NH_3_ for 6 h at 723 K.

Figure 10 shows the grazing incidence XRD patterns obtained from the Pd-Ru membrane after exposure to 10% NH_3_ at 723 K for 6 h. After the NH_3_ exposure, there is no significant change in the phase of the Pd-Ru membrane, which is consistent with the fresh membrane.

**Figure 11:**
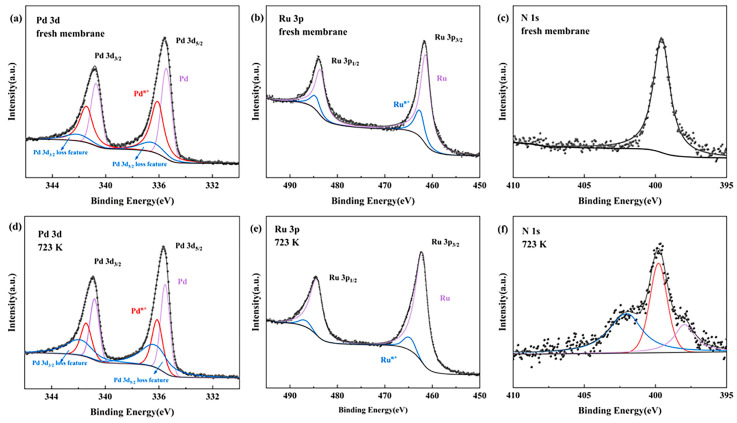
High-resolution XPS spectrum of Pd 3d, Ru 3p, and N 1s signals for Pd-Ru membranes. (**a**–**c**) Fresh membrane; (**d**–**f**) membrane after exposure to 10% NH_3_ at 723 K.

Figure 11 shows the high-resolution XPS spectrum of Pd 3d, Ru 3p, and N 1s signals for the Pd-Ru membranes before and after exposure to 10% NH_3_ at 723 K for 6 h. All three elements, i.e., Pd, Ru, and N, are detected on the surface of the membrane. There are contributions from high valence states of Pd^n+^ and Ru^n+^ [32]. Figure 11c shows that a fresh Pd-Ru membrane surface results in a dominant N 1s peak centered at an energy level of 399.8 eV corresponding to NH_3_, which may be introduced by the process of electroless plating. When the Pd-Ru membrane is exposed to 10% NH_3_ at 723 K, the N 1s spectrum is rather broad and exhibits at least three distinct peaks. It can be seen that the fitted N 1s spectrum reveals a main peak at around 399.8 eV, corresponding to NH_3_, and the peak at 397.8 eV is assigned to NH_X_ (x = 1, 2) [33]. The N 1s spectrum at 402 eV could not be identified. During NH_3_ exposure, NH_3_ molecules and other nitrogenous species are adsorbed on the surface of the Pd-Ru membrane, covering the hydrogen adsorption sites and thus inhibiting hydrogen permeation through the Pd-Ru membrane.

Ru is the catalyst for the cracking of NH_3_. NH_3_ consecutively decomposes as NH_2_ → NH → N on the active site. During NH_3_ decomposition, NH_3_ is readily dissociated to give NH_2_ and NH_2_ is easily fragmented to form NH. When the Pd-Ru membrane is exposed to NH_3_, a portion of the active site becomes covered in NH_3_, whereas a small amount of NH_3_ decomposition should result in a certain coverage of NH_X_ species.

**Figure 12:**
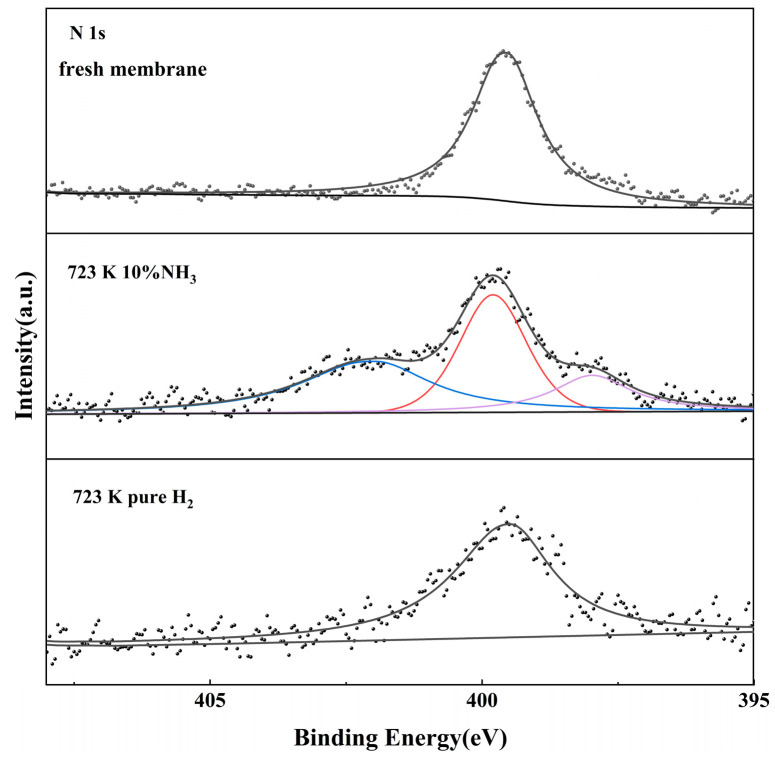
High-resolution XPS spectrum of N 1s signals for Pd-Ru membranes after different treatments.

Figure 12 shows the XPS spectrum of the N 1s for Pd-Ru membranes after different treatments. Compared with the Pd-Ru membrane exposed to NH_3_, the species in the 397.8 eV and 402 eV range disappeared after pure hydrogen treatment, indicating that only physical adsorption but not chemical adsorption occurred after NH_3_ exposure. When pure hydrogen was reintroduced, the dissociation and adsorption equilibrium of NH_3_ on the membrane surface moved in the opposite direction, resulting in the almost complete desorption of physically adsorbed NH_3_ and NH_x_ (x = 1, 2). Therefore, the hydrogen fluxes all recovered 100% after pure hydrogen treatment.

The combination of the hydrogen permeation test, SEM, XRD, and XPS results suggests that physical adsorption and concentration polarization of nitrogenous species occur simultaneously when the Pd-Ru membrane is exposed to NH_3_. The competitive adsorption effect weakens and the extent of the concentration polarization effect increases with the temperature rising. No additional defects are formed on the Pd-Ru membrane during NH_3_ exposure, and the deactivations of the membranes are reversible after pure hydrogen is introduced. When Pd-Ru membranes are exposed to NH_3_, the effective area decreases due to the adsorption of the nitrogenous species on the surfaces, and thus the hydrogen flux decreases. Pure hydrogen treatment results in almost complete desorption of physically adsorbed NH_3_ and NH_x_ (x = 1,2) and recovery of hydrogen flux. Otherwise, the high NH_3_ concentration or the long exposure time may promote a diffusion of the Pd to the surface and thus enhance the adsorption of nitrogenous species. The enrichment of Ru on the membrane surface may have a unique effect on the NH_3_ tolerance of the Pd-Ru membrane.

## 4. Conclusions

In this study, we investigated the effect of NH_3_ concentration and temperature on the hydrogen flux and subsequent recovery in pure hydrogen of the Pd-Ru membranes. The results showed that Pd-Ru membranes exhibit excellent NH_3_ tolerance. The following results were observed:

(1) The hydrogen flux of the Pd-Ru membrane remains 67–85% under exposure to 1–10% NH_3_ for 6 h at 723 K. A higher NH_3_ concentration leads to greater inhibition of hydrogen flux, which can be fully recovered after the removal of NH_3_.
(2) The inhibition effect of NH_3_ increases with the temperature increasing. The hydrogen flux of Pd-Ru membranes rapidly decreases by 27–50% after 10% NH_3_ exposure at 673–773 K.
(3) A difference in relative hydrogen flux of 5.9% is observed at 773 K between 10% NH_3_ and 10% N_2_ exposure, while a difference of 13.3% is observed at 673 K. The poisoning effect of NH_3_ on Pd-Ru membranes is attributed to both competitive adsorption and concentration polarization. With increasing temperature, the competitive adsorption effect of NH_3_ decreases and the concentration polarization effect increases.
(4) The XPS analysis of the membranes after exposure to NH_3_ showed that the reduction in hydrogen flux is attributed to the decrease in effective area due to the adsorption of the nitrogenous species on the surfaces during the dissociation process of hydrogen molecules. The poisoning effect of NH_3_ on Pd-Ru membranes is completely reversible.

## Figures and Tables

**Table 1:** Bath compositions of Pd-Ru electroless plating.

Chemicals	Pd Bath	Ru Bath
PdCl_2_	4.36 g/L	/
RuCl_3_	/	0.0178 g/L
EDTA	60 g/L	/
NH_3_∙H_2_O	600 mL/L	150 mL/L
N_2_H_4_	1 vol%	10 vol%
Temperature	308 K	333 K

## Data Availability

The data presented in this study are available on request from the corresponding authors due to restrictions, e.g., privacy or ethical.
